# CMV Detection and Pneumocystis Infection in the PERCH Cohort

**DOI:** 10.3390/jof12100761

**Published:** 2026-10-10

**Authors:** Dora Pungan, Ava Wasserstein, Mst Shamima Khatun, Haiyan Miller, Jerry S. Zifodya, Jay K. Kolls

**Affiliations:** 1 Center for Translational Research in Infection and Inflammation, John W Deming Department of Internal Medicine, School of Medicine, Tulane University, New Orleans, LA 70112, USA; dpungan@tulane.edu (D.P.); awasserstein@tulane.edu (A.W.); mkhatun@tulane.edu (M.S.K.); hdeng@tulane.edu (H.M.); jzifodya@tulane.edu (J.S.Z.); 2 Departments of Medicine and Pediatrics, School of Medicine, Tulane University, New Orleans, LA 70112, USA

**Keywords:** *Pneumocystis*, Cytomegalovirus, pneumonia

## Abstract

The Pneumonia Etiology Research for Child Health (PERCH) study identified pneumonia due to the fungus *Pneumocystis jirovecii* as the most common fungal cause of pneumonia in infants. Whether *Pneumocystis jirovecii* pneumonia (PJP) is complicated by other pathogens remains unclear. To address this, we performed targeted next-generation sequencing on archived PERCH samples and found that human cytomegalovirus (CMV) was the most common virus detected, particularly in Africa. Here, CMV status was defined as detection of CMV nucleic acid in the nasopharyngeal specimen by targeted sequencing, not CMV seropositivity. Despite CMV detection being common, analyses of clinical metadata did not show any impact on clinical parameters such as oxygen requirement or hospital stay. Because sequence detection reflects the presence of CMV at the upper respiratory tract, these findings should not be interpreted as evidence of a causal role in pneumonia. However, future studies should consider assessing CMV co-infection to determine if CMV affects post-pneumonia sequelae in children.

## 1. Introduction

The Pneumonia Etiology Research for Child Health (PERCH) study was a large, multi-country case–control study designed to identify the causes of severe pneumonia among children aged 1–59 months in low- and middle-income settings, using standardized clinical assessments and microbiologic testing across sites in Africa and Asia. A central component of PERCH was the systematic collection of nasopharyngeal and oropharyngeal (NP-OP) swabs from both cases and community controls, enabling molecular detection of respiratory pathogens by multiplex PCR. Using these NP-OP specimens, PERCH identified *Pneumocystis jirovecii* as significantly associated with pneumonia, with a greater etiologic contribution observed among younger infants, consistent with known age-related susceptibility to *Pneumocystis* infection [1].

In our study, we obtained archived nucleic acid samples from NP-OP swabs from the PERCH cohort to further investigate the epidemiology of *P. jirovecii* in infants and young children with severe pneumonia and study viral fungal interactions. We first surveyed the broader spectrum of respiratory pathogens in this population, using a targeted respiratory pathogen assay and CMV was the predominant viral co-pathogen identified, and it therefore became the primary focus of the secondary analyses that followed.

Most evidence linking *Pneumocystis jirovecii* pneumonia (PJP) to Cytomegalovirus (CMV) co-infection derives from immunocompromised adults. A systematic review and meta-analysis of adult kidney transplant recipients identified CMV infection as a risk factor for PJP, although the pooled studies varied in their PJP diagnostic criteria [2]. In a targeted next-generation sequencing study of adult patients with severe pneumonia stratified by CD4^+^ T-cell count, detection rates of both PJP and CMV increased in parallel with worsening cellular immunosuppression, with both pathogens most prevalent at CD4^+^ counts <100 cells/µL [3]. Similarly, a metagenomic analysis of non-HIV-immunocompromised adults found CMV among the most frequently detected co-pathogens in bronchoalveolar lavage from patients with PJP, occurring significantly more often than in non-PJP-type pneumonia [4]. By contrast, whether PJP-CMV co-infection occurs in young children—the majority of whom are not overtly immunosuppressed—remains poorly defined. The PERCH cohort enrolled infants and children with pneumonia who were largely HIV-negative and without a documented immunocompromising condition, and it is in this population that we examined CMV detection.

Unlike a bystander viral infection, CMV encodes its own repertoire of immunomodulatory proteins, including homologs of human cytokines and cytokine/chemokine receptors [5]. Experimentally, in the context of *Aspergillus fumigatus*, CMV suppresses antifungal effector responses in co-infection models, although this appears to operate through multiple mechanisms rather than the viral IL-10 homolog alone [6]. Together these provide a plausible mechanistic rationale for CMV–PJP co-occurrence.

Because PJP may occur alongside viral or polymicrobial co-infection, a sensitive and comprehensive approach was needed to assess potential co-pathogens in PERCH NP-OP specimens. We therefore used Illumina’s Respiratory Pathogen ID/AMR (RPIP) Enrichment Kit, which enhances detection of a broad range of respiratory viruses, bacteria, and fungi from low-biomass samples. This targeted enrichment strategy allowed systematic evaluation of co-infections among PJP-positive cases within the PERCH cohort.

## 2. Materials and Methods

### 2.1. Sample Selection

Samples were obtained from the PERCH cohort. Enrolled children were predominantly HIV-negative and had no documented immunocompromising condition. The PERCH clinical dataset includes detailed demographic, clinical, and laboratory information for enrolled participants. All specimens were archived nasopharyngeal (NP) swabs stored at –80 °C. To study co-infection, extracted nucleic acids were used for Rapid Pathogen Identification Panel (RPIP, Illumina, San Diego, CA, USA) with or without Whole Transcriptome Amplification (WTA) as outlined below.

For this project, we performed a preliminary pilot analysis with RPIP in PJP+ and PJP− cases to assess co-infection prevalence. The pilot analysis identified an association between CMV positivity and PJP status, particularly in samples from Africa, motivating expansion to a larger validation cohort. Based on pilot effect estimates, we performed RPIP on an additional forty PERCH specimens. We restricted our analysis to samples collected in Mali and Zambia, where prior internal pilot work demonstrated a higher prevalence of CMV-PJP co-infection. This restriction ensured an adequate number of positive cases for a powered analysis but may inflate the apparent co-infection prevalence and limits generalizability to the full multi-country cohort (see Section 4, Discussion). All samples selected for testing had been previously classified as PJP positive (PJP+) according to the PERCH diagnostic algorithm. Clinical metadata used for downstream analyses included lowest recorded oxygen saturation, length of hospital stay, HIV status, and additional severity metrics available within the PERCH database.

### 2.2. Whole Transcriptome Amplification (WTA)

PERCH NP swab specimens contain low-input nucleic acid biomass, so Whole Transcriptome Amplification (WTA) was performed to generate sufficient cDNA for downstream pathogen detection. WTA was performed using the REPLI-G WTA Kit (Qiagen, Germantown, MD, USA) according to the manufacturer’s recommendations. Briefly, total RNA was first reverse-transcribed to generate cDNA using random hexamer priming. The resulting cDNA underwent isothermal linear amplification using phi29 DNA polymerase, a high-fidelity strand-displacing enzyme characteristic of the REPLI-G platform. Isothermal amplification was conducted at a constant temperature of 30 °C for several hours, producing a uniformly amplified cDNA pool with minimal sequence bias, consistent with previously described applications of REPLI-G technologies. Amplified cDNA was purified, quantified using fluorometric measurement, and assessed for fragment integrity. Only samples meeting predefined quality thresholds were advanced to RPIP testing. Amplified cDNA was stored at −20 °C prior to pathogen panel analysis.

### 2.3. Rapid Pathogen Identification Panel (RPIP)

Pathogen detection was performed using an Illumina RNA Prep with Enrichment workflow applied to the amplified cDNA generated from WTA. The full RPIP pathogen list is listed in the data supplement. Beginning at the Clean-Up cDNA step of the manufacturer’s protocol (Illumina, San Diego, CA, USA), amplified cDNA was purified using magnetic beads to remove residual primers, enzymes, and short fragments, then eluted and quantified before library preparation. The purified cDNA was then subjected to tagmentation using Enrichment Bead-Linked Transposomes, which simultaneously fragmented double-stranded cDNA and added adapter sequences required for downstream amplification. Tagmented fragments were purified and amplified through limited-cycle PCR to incorporate dual index adapters and P5/P7 flow-cell sequences. The resulting libraries were magnetically bead-purified to remove small byproducts and adapter dimers. Following amplification, libraries were quantified and normalized using bead-based normalization reagents and subsequently pooled at equimolar concentrations, consistent with manufacturer-recommended inputs (200 ng per library). The pooled libraries underwent probe hybridization using an Illumina enrichment panel targeting predefined viral, bacterial, and fungal genomic regions. Hybridized fragments were captured with magnetic beads, washed at elevated temperatures to remove nonspecific binding, and eluted as enriched libraries. Enriched pools were then subjected to a 14-cycle PCR amplification to increase yield and to finalize sequencing constructs. A final magnetic bead purification step removed residual primers and reaction components. Library concentration and fragment-size distribution were assessed using the Qubit dsDNA HS Assay (Thermo Fisher Waltham, MA, USA) and Agilent Tapestation 4150 with DNA1000 kit (Agilent Technologie, Santa Clara, CA, USA) to confirm suitability for sequencing. Sequencing was performed on an Illumina platform using paired-end chemistry according to manufacturer-recommended run parameters. Sequencing reads were processed in BaseSpace using the DRAGEN Microbial Enrichment Plus, V 1.1.1, which performed demultiplexing, adapter trimming, quality filtering, and alignment to curated pathogen reference databases. Pathogen detection was qualitative, with each organism reported as present or absent based on internal thresholding. CMV was detected through the viral panel, and *Pneumocystis jirovecii* through the fungal panel. Internal positive and negative controls performed as expected, verifying assay validity. For all analyses, a sample was classified as CMV co-infected when CMV nucleic acid was detected in the NP swab by the RPIP viral panel (a qualitative present/absent call); viral load was not quantified and CMV serostatus was not assessed.

### 2.4. Statistical Analysis

Prevalence of CMV co-detection with PJP was compared using Fisher’s exact test, given the small cell counts in region-stratified analyses. For the clinical metadata, data are presented as median (interquartile range [IQR]) for continuous variables or a number (percentage) for categorical variables. Differences in baseline characteristics between groups were assessed via Wilcoxon rank-sum test or χ^2^ test, respectively. For sample-size estimation only, tachypnea was used as the anchoring covariate; as it is a sensitive indicator of lung disease in infants, it was among the most consistently recorded clinical variables, and was associated with CMV positivity in the pilot data.

## 3. Results

### 3.1. Results of the Initial Pilot Study

We initially performed RPIP on 50 random PERCH samples spanning the study sites (Table 1).

The only significant bacterial co-infection was Klebsiella pneumoniae, detected in 43% (6/14) of African PJP+ samples versus 0% (0/12) of African PJP− samples (*p* = 0.017), with no corresponding difference in the Asian cohort (Table 2). Among viruses, rhinoviruses and other respiratory viruses were detected sporadically and comparably across groups, and CMV was the only virus that differed by PJP status (Table 2). Fungal co-detections other than *P. jirovecii* were rare, and limited to *Fusarium oxysporum* (2 samples) and *Rasamsonia aegroticola* (1 sample), with no difference by PJP status.

In Table 2, values are the number of positive samples (percentage of subgroup). *p* compares PJP+ vs. PJP− within each region by Fisher exact test; bold indicates *p* < 0.05. Fungal co-detections other than *P. jirovecii* were rare: *Fusarium oxysporum* (2 samples) and *Rasamsonia aegroticola* (1 sample), with no difference by PJP status. Rare bacterial pathogens (<3 detections) are omitted from the table and are listed here: *Acinetobacter baumannii*, *Actinomyces graevenitzii*, *Capnocytophaga gingivalis*, *Capnocytophaga leadbetteri*, *Capnocytophaga sputigena*, *Enterobacter cloacae complex*, *Haemophilus haemolyticus*, *Haemophilus parahaemolyticus*, *Kingella kingae*, *Neisseria lactamica*, *Proteus mirabilis*, *Pseudomonas aeruginosa*, *Pseudomonas fluorescens*, *Streptococcus constellatus*, *Streptococcus dysgalactiae*, *Tropheryma whipplei*, *Ureaplasma parvum*, *Veillonella parvula*. Rare viral pathogens (<3 detections, omitted): *Human Parainfluenza virus 1*, *Human adenovirus B*, *Human bocavirus 1*, *Human coronavirus NL63*, *Human metapneumovirus*, *Human parainfluenza virus 2*, *Influenza A virus (H3N2)*, *Respiratory syncytial virus A*.

**Figure 1:**
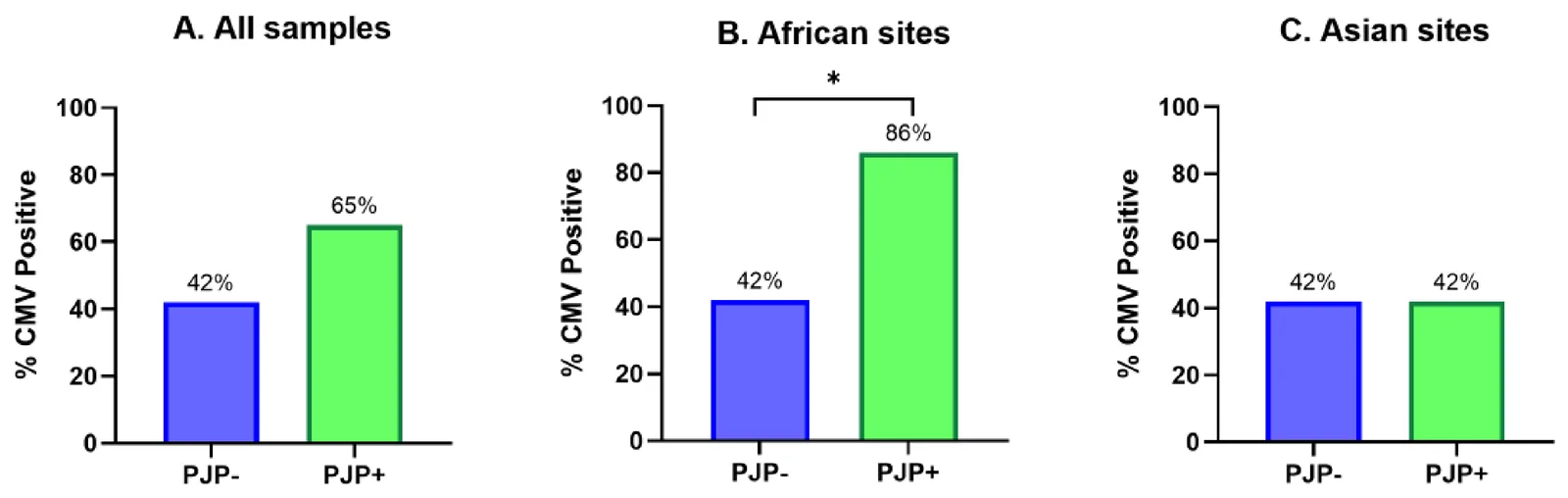
Association between Cytomegalovirus (CMV) and *Pneumocystis jirovecii* pneumonia (PJP) co-detection. (**A**) Pooled analysis of all pilot samples, shown for completeness; CMV was detected in 17/26 (65%) of PJP-positive versus 10/24 (42%) of PJP-negative samples (Fisher’s exact *p* = 0.16). (**B**) Stratifying by region revealed that the association was confined to the African sites (Mali and Zambia), where CMV was present in 12/14 (86%) of PJP-positive versus 5/12 (42%) of PJP-negative samples (* denotes Fisher’s exact *p* = 0.038). (**C**) No association was observed at the Asian sites (Bangladesh and Thailand), where CMV positivity was identical between groups (5/12, 42% in both; *p* = 1.0).

Across the full pilot cohort, CMV was detected more often in PJP+ than PJP− samples (17/26, 65% vs. 10/24, 42%), but this pooled comparison did not reach statistical significance (Fisher’s exact *p* = 0.16) and combines two geographically distinct regions. Stratified analysis showed that the association was confined to the African sites (Figure 1B) and absent at the Asian sites (Figure 1C).

Because HIV-positive children may be susceptible to both pathogens for reasons unrelated to viral–fungal interaction, we repeated the African comparison after excluding the five HIV-positive samples. The magnitude of the association was essentially unchanged (PJP+ 9/11, 82% vs. PJP− 4/10, 40%), although with the reduced sample size it no longer reached statistical significance (Fisher’s exact *p* = 0.08).

### 3.2. Effect of CMV Detection and Clinical Metadata in PERCH

As infant CMV rates are known to be high in Africa, we conducted a second study confined to PERCH samples from Africa to determine if CMV had any effect on the clinical course of PJP infection. To ensure adequate statistical power to validate this association in a larger cohort, we performed a sample size calculation using tachypnea as a representative clinical covariate associated with CMV positivity (used only to anchor the effect-size estimate, not as a study outcome). Sample size calculations using standard formulas for dichotomous outcomes (alpha = 0.05, power = 80%), with reference to the ClinCalc online calculator, indicated that a minimum of 40 samples would be required to detect a meaningful association. Accordingly, the validation cohort was expanded to include 40 PJP+ specimens from Mali and Zambia (Supplemental Table S1), previously identified as sites with a higher frequency of CMV–PJP co-infection. Of 40 specimens sequenced in the validation batch, one was excluded owing to failed enrichment, with no organisms detected across all three panels, and three specimens had already been analyzed in the discovery cohort, yielding 36 unique validation specimens.

**Figure 2:**
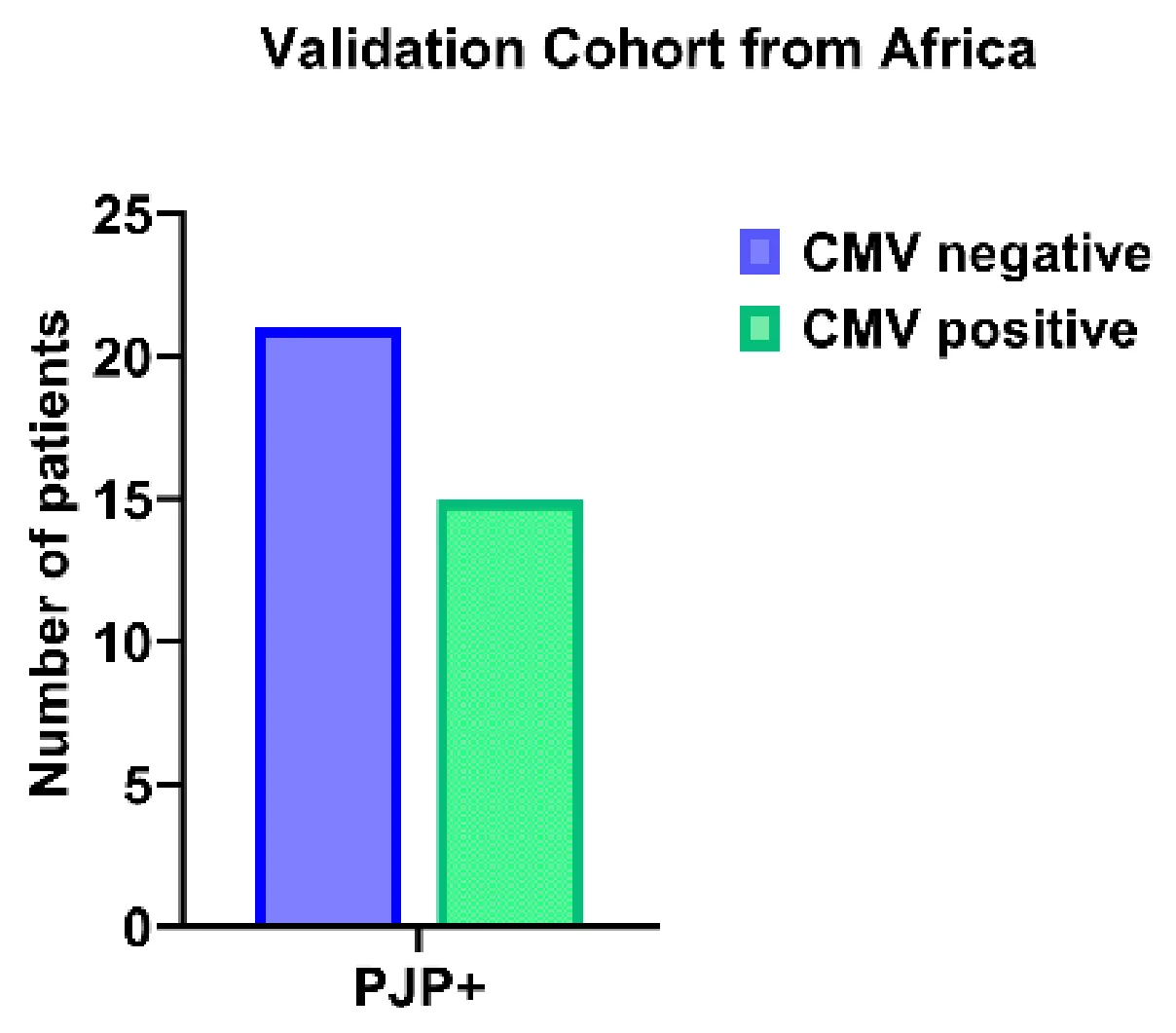
CMV prevalence in the validation cohort as measured by RPIP.

CMV was detected in 15 of 36 specimens (41.7%, Figure 2). Positivity did not differ between the two African sites (Mali 7/19, 36.8% vs. Zambia 8/17, 47.1%; Fisher’s exact *p* = 0.74), and specimens were therefore analyzed as a pooled African cohort. HIV status was negative in 25 specimens and unconfirmed in 11, with no HIV-positive specimens; restricting the analysis to HIV-negative specimens yielded a nearly identical CMV positivity of 40.0% (10/25). Because the validation cohort was restricted to PJP-positive specimens, it lacks the PJP-negative comparison group required to test the pilot association (Figure 1) and instead characterizes CMV within PJP-positive cases.

### 3.3. Demographics and Clinical Parameters in PJP-CMV Co-Detection in Samples for Africa

We next aggregated data for all samples from Africa, including the discovery cohort. Thus, for the clinical comparisons in Table 3, data from the pilot (n = 29) and validation (n = 36) cohorts were pooled. The three specimens sequenced in both cohorts were counted once, and one validation specimen was excluded due to missing data, yielding 86 unique samples (34 CMV-positive, 31 CCMV-negative). Consistent with the parent PERCH study, the cohort consisted predominantly of infants, with a median age of 3 months in both groups. CMV-positive samples were slightly older than CMV-negative, with the difference reflected in the interquartile range. Both groups had similar rates of HIV exposure and PERCH-defined PJP positivity. After excluding the five HIV-positive cases, 60 samples remained for the sensitivity analysis, in which the CMV age association was unchanged in direction and magnitude, although it no longer reached statistical significance (*p* = 0.05). There was no difference in hospital length of stay or level of oxygen support. Nutritional status as measured by weight or Mid-Upper Arm Circumference (MUAC) did not differ, although MUAC data were only available for 41 samples.

In Table 3, the data represent all unique specimens from the discovery and validation cohorts combined (n = 86). Continuous variables are presented as median (interquartile range) and compared using the Wilcoxon rank-sum test; categorical variables are presented as number (percentage) and compared using the χ^2^ test. PJP and HIV status are as classified by the PERCH study. Denominators vary by characteristic owing to incomplete clinical metadata, and the number of specimens with available data is indicated where it differs from the full cohort. MUAC, mid-upper arm circumference.

## 4. Discussion

Using a targeted next-generation sequencing platform on archived nucleic acid samples derived from NP-OP swabs from PERCH, we found that human CMV was commonly detected in PJP+ samples, particularly in sub-Saharan Africa. Because CMV was defined by qualitative nucleic acid detection in an upper-respiratory specimen, these findings reflect the presence of CMV in the nasopharynx and cannot distinguish latent shedding from active disease, nor establish a causal role in pneumonia. The higher prevalence in African infants is consistent with prior prevalence studies showing high rates of CMV there [7]. In prior studies of human PJP, CMV has also been described [8]. In one retrospective study of HIV+ PJP, CMV positivity was associated with increased odds of both in-hospital mortality and mortality at one year [9]. Another retrospective study in renal transplant recipients who developed PJP noted that CMV co-infection was associated with increased renal dysfunction/graft failure [10]. Perret et al. conducted a retrospective study of both HIV-associated and non-HIV-associated cases of PJP, and CMV was detected in the lung lavage in 25% of the cases [10]. In this study, CMV viral load was associated with an increased requirement of intensive care. The authors state that it is unclear if CMV infection is altering the pathogenesis of PJP or a marker of a greater immunocompromised state.

In our study, CMV detection was prevalent in infants, but we did not detect any differences in oxygen requirement or length of stay. It is of interest that a study of African infants found that CMV infection did not impair vaccine-specific responses following diphtheria–tetanus–pertussis or measles vaccination [11], suggesting that CMV infection in this population does not broadly compromise adaptive immune responses to childhood vaccines.

Our study has limitations. Detection reflects upper-respiratory nucleic acid rather than lower-respiratory disease, and the qualitative assay does not quantify viral load. The validation cohort was deliberately restricted to Mali and Zambia, the sites with the highest CMV–PJP co-detection in the pilot analysis. This design ensured an adequate number of positive cases for the planned comparison, but it selects on the exposure of interest and therefore inflates the apparent co-infection prevalence relative to the full multi-country PERCH cohort. The validation prevalence should not be read as a generalizable estimate. Restriction to PJP-positive specimens further removed the PJP-negative comparison group on which the pilot association depended, so the validation cohort characterizes CMV prevalence within PJP-positive cases rather than testing that association. The two cohorts were also processed and sequenced approximately one year apart as separate batches, introducing potential batch effects in library preparation and sequencing that cannot be separated from cohort differences. Taken together, our findings should be regarded as hypothesis-generating.

## 5. Conclusions/Future Directions

This study shows that CMV detection is common in infant cases of PJP but we did not detect any clinical sequelae in terms of the acute infection. Longitudinal studies would be needed to determine if there are any long-term sequelae in terms of lung function or other pulmonary complications in the context of CMV co-infection.

## Figures and Tables

**Table 1:** Demographics table of samples analyzed in this pilot study.

Sample ID_PERCH	Enrollment Site	Age (Months)	Sex	HIV Status	PJP Status_PERCH
M03122	Mali	5	female	positive	positive above threshold
M05012	Mali	4	male	unknown	positive above threshold
D00373	Bangladesh	3	male	negative	positive above threshold
M01120	Mali	2	male	negative	positive above threshold
M00900	Mali	4	male	negative	positive above threshold
Z02654	Zambia	6	male	negative	positive above threshold
Z02151	Zambia	3	female	positive	positive above threshold
Z02097	Zambia	3	female	negative	positive above threshold
Z01427	Zambia	3	female	negative	positive above threshold
Z01404	Zambia	3	female	positive	positive above threshold
M04570	Mali	2	male	unknown	positive above threshold
M05512	Mali	3	male	unknown	positive above threshold
M03593	Mali	3	female	unknown	positive above threshold
Z02172	Zambia	4	female	negative	positive above threshold
D00187-39	Bangladesh	3	female	negative	positive above threshold
D00480-39	Bangladesh	8	male	negative	positive above threshold
D00492-39	Bangladesh	3	male	negative	positive above threshold
B00443	Bangladesh	4	male	negative	positive above threshold
B00056	Bangladesh	3	male	negative	positive above threshold
T15254	Thailand	3	female	negative	positive above threshold
N13909	Thailand	3	female	negative	positive above threshold
T10281	Thailand	2	female	negative	positive above threshold
N17953	Thailand	2	female	negative	positive above threshold
N17354	Thailand	2	female	negative	positive above threshold
T15253	Thailand	2	male	negative	positive above threshold
B00452	Bangladesh	1	female	negative	negative
Z02078	Zambia	5	female	negative	negative
B00204	Bangladesh	3	male	negative	negative
N14007	Thailand	4	male	negative	negative
M02170	Mali	2	male	unknown	positive above threshold
Z00022	Zambia	19	male	positive	negative
Z02900	Zambia	2	male	negative	negative
Z00305	Zambia	1	male	negative	negative
Z00815	Zambia	5	female	positive	negative
M00157	Mali	3	male	negative	negative
M02959	Mali	5	male	unknown	negative
M02743	Mali	7	female	unknown	negative
M02594	Mali	4	male	unknown	negative
M02542	Mali	0	male	unknown	negative
M03427	Mali	2	female	negative	negative
Z03343	Zambia	1	female	negative	negative
B00043	Bangladesh	2	male	negative	negative
D00512	Bangladesh	2	male	negative	negative
D00515	Bangladesh	2	female	negative	negative
D00474	Bangladesh	3	female	negative	negative
T15831	Thailand	5	male	negative	negative
T11012	Thailand	3	male	negative	negative
N15298	Thailand	21	female	negative	negative
N16207	Thailand	5	female	negative	negative
T13812	Thailand	5	female	negative	negative

**Table 2:** Co-Infection Patterns of Bacterial and Viral Pathogens with *Pneumocystis jirovecii* Pneumonia (PJP).

Organism	African Samples (n = 26)			Asian Samples (n = 24)		
	PJP+ (n = 14)	PJP− (n = 12)	*p*	PJP+ (n = 12)	PJP− (n = 12)	*p*
**Bacteria**						
*Rothia mucilaginosa*	10 (71%)	10 (83%)	0.652	7 (58%)	11 (92%)	0.155
*Moraxella catarrhalis*	10 (71%)	8 (67%)	1.000	5 (42%)	9 (75%)	0.214
*Streptococcus pneumoniae*	10 (71%)	10 (83%)	0.652	5 (42%)	7 (58%)	0.684
*Dolosigranulum pigrum*	11 (79%)	7 (58%)	0.401	5 (42%)	5 (42%)	1.000
*Haemophilus influenzae*	7 (50%)	5 (42%)	0.713	4 (33%)	3 (25%)	1.000
*Gemella haemolysans*	5 (36%)	5 (42%)	1.000	2 (17%)	6 (50%)	0.193
*Streptococcus mitis*	4 (29%)	1 (8%)	0.330	4 (33%)	5 (42%)	1.000
*Corynebacterium propinquum*	4 (29%)	4 (33%)	1.000	2 (17%)	1 (8%)	1.000
*Corynebacterium pseudodiphtheriticum*	6 (43%)	2 (17%)	0.216	1 (8%)	1 (8%)	1.000
*Prevotella melaninogenica*	2 (14%)	4 (33%)	0.365	1 (8%)	2 (17%)	1.000
*Staphylococcus aureus*	2 (14%)	3 (25%)	0.635	3 (25%)	1 (8%)	0.590
*Klebsiella pneumoniae*	6 (43%)	0 (0%)	**0.017**	1 (8%)	1 (8%)	1.000
*Actinomyces odontolyticus*	2 (14%)	1 (8%)	1.000	0 (0%)	1 (8%)	1.000
*Haemophilus parainfluenzae*	1 (7%)	1 (8%)	1.000	0 (0%)	2 (17%)	0.478
*Neisseria meningitidis*	1 (7%)	1 (8%)	1.000	0 (0%)	2 (17%)	0.478
*Neisseria flavescens*	1 (7%)	1 (8%)	1.000	0 (0%)	1 (8%)	1.000
**Viruses**						
Cytomegalovirus (CMV)	12 (86%)	5 (42%)	**0.038**	5 (42%)	5 (42%)	1.000
Human rhinovirus A	6 (43%)	4 (33%)	0.701	4 (33%)	4 (33%)	1.000
Human rhinovirus C	4 (29%)	6 (50%)	0.422	3 (25%)	3 (25%)	1.000
Respiratory syncytial virus B	3 (21%)	1 (8%)	0.598	2 (17%)	2 (17%)	1.000
Epstein–Barr virus (EBV)	2 (14%)	2 (17%)	1.000	2 (17%)	0 (0%)	0.478
Human rhinovirus B	2 (14%)	0 (0%)	0.483	2 (17%)	1 (8%)	1.000
Human herpesvirus 6 (HHV-6)	1 (7%)	0 (0%)	1.000	1 (8%)	2 (17%)	1.000
Human parainfluenza virus 3	2 (14%)	1 (8%)	1.000	0 (0%)	0 (0%)	1.000

**Table 3:** Clinical metadata table of all samples analyzed by RPIP (pooled pilot and validation cohorts, n = 65), stratified by CMV detection.

Characteristic	CMV Positive (n = 34)	CMV Negative (n = 31)	*p*-Value
Age (months)	3.0 (2.3, 4.0)	3.0 (2.0, 3.0)	0.02
Male sex	19 (56%)	16 (52%)	0.92
PJP positive	29 (85%)	24 (77%)	0.62
HIV positive (available for n = 44)	4 (17%)	1 (4.8%)	0.40
HIV exposed (available for n = 45)	12 (48%)	6 (30%)	0.36
Oxygen saturation (%) (available for n = 45)	85 (80, 94)	89 (83, 94)	0.55
Oxygen support (ever) (available for n = 45)	17 (74%)	12 (55%)	0.29
Respiratory rate (breaths/min) (available for n = 64)	57 (44, 72)	61 (53, 71)	0.46
Length of stay (days) (available for n = 45)	4.0 (2.0, 12)	4.0 (1.3, 8.5)	0.71
MUAC-for-age (z-score) (available for n = 41)	−1.7 (−2.4, −0.34)	−0.68 (−2.5, 0.10)	0.36
Weight-for-age (z-score)	−2.0 (−3.0, −0.70)	−0.77 (−2.0, −0.23)	0.05
Alive at 30 days (available for n = 36)	6 (33%)	10 (56%)	0.31
Temperature (C) (available for n = 59)	37 (36, 38)	37 (36, 38)	0.40
Illness duration (days) (available for n = 47)	7.0 (2.0, 9.5)	4.0 (3.0, 7.0)	0.35

## Data Availability

RPIP data are included in the Supplemental Data.

## Supplementary Materials

The following supporting information can be downloaded at: https://www.mdpi.com/article/10.3390/jof12100761/s1, Table S1: Demographics table of samples analyzed in the African validation study; RPIP pathogen list; RPIP results.

## Abbreviations

The following abbreviations are used in this manuscript:

PJP	*Pneumocystis jirovecii* Pneumonia
CMV	Cytomegalovirus
