# Supplementary material for: CMV Detection and Pneumocystis Infection in the PERCH Cohort
Source: J Fungi (Basel). 2026 Oct 10;12(10):761. doi: 10.3390/jof12100761 (PMC13706021; doi:10.3390/jof12100761)
Supplement: Supplementary file 1 [file jof-12-00761-s001.zip › Supplemental Data-Table S1.pdf]

Supplemental Data

Supplemental Table S1: **Demographics table of samples analyzed in the African validation study.**

Sample ID_PERCH	Enrollment Site	Age (months)	Sex	HIV Status	PJP status_PERCH
M00272	Mali	2	female	unknown	Pjp positive above threshold
M01382	Mali	2	male	negative	Pjp positive above threshold
M01949	Mali	4	male	negative	Pjp positive above threshold
M02279	Mali	4	female	unknown	Pjp positive above threshold
M02413	Mali	3	female	unknown	Pjp positive above threshold
M02460	Mali	3	male	negative	Pjp positive above threshold
M02752	Mali	3	female	unknown	Pjp positive above threshold
M02981	Mali	3	female	negative	Pjp positive above threshold
M03329	Mali	3	male	unknown	Pjp positive above threshold
M03486	Mali	3	male	negative	Pjp positive above threshold
M03521	Mali	2	female	unknown	Pjp positive above threshold
M03593	Mali	3	female	negative	Pjp positive above threshold
M03939	Mali	2	male	negative	Pjp positive above threshold
M03983	Mali	5	female	negative	Pjp positive above threshold

M04189	Mali	2	female	unknown	Pjp positive above threshold
M04506	Mali	3	female	unknown	Pjp positive above threshold
M04570	Mali	2	male	unknown	Pjp positive above threshold
M05037	Mali	3	male	unknown	Pjp positive above threshold
M05309	Mali	3	female	unknown	Pjp positive above threshold
M05371	Mali	4	female	negative	Pjp positive above threshold
M05512	Mali	3	male	unknown	Pjp positive above threshold
M05678	Mali	3	male	unknown	Pjp positive above threshold
Z00027	Zambia	2	male	negative	Pjp positive above threshold
Z00456	Zambia	2	male	negative	Pjp positive above threshold
Z00814	Zambia	6	female	negative	Pjp positive above threshold
Z00897	Zambia	3	male	negative	Pjp positive above threshold
Z01108	Zambia	2	male	negative	Pjp positive above threshold
Z01382	Zambia	3	female	negative	Pjp positive above threshold
Z01742	Zambia	2	male	negative	Pjp positive above threshold
Z01771	Zambia	2	female	negative	Pjp positive above threshold
Z01936	Zambia	3	female	negative	Pjp positive above threshold

Z02245	Zambia	2	male	negative	Pjp positive above threshold
Z02348	Zambia	2	male	negative	Pjp positive above threshold
Z02361	Zambia	2	female	negative	Pjp positive above threshold
Z02456	Zambia	2	male	negative	Pjp positive above threshold
Z02661	Zambia	3	male	negative	Pjp positive above threshold
Z03040	Zambia	4	male	negative	Pjp positive above threshold
Z03130	Zambia	3	male	negative	Pjp positive above threshold
Z03364	Zambia	2	female	negative	Pjp positive above threshold
